# Skeletal Muscle Contractions Induce Acute Changes in Cytosolic Superoxide, but Slower Responses in Mitochondrial Superoxide and Cellular Hydrogen Peroxide

**DOI:** 10.1371/journal.pone.0096378

**Published:** 2014-05-29

**Authors:** Timothy Pearson, Tabitha Kabayo, Rainer Ng, Jeffrey Chamberlain, Anne McArdle, Malcolm J. Jackson

**Affiliations:** 1 Institute of Ageing and Chronic Disease, University of Liverpool, Liverpool, United Kingdom; 2 Department of Neurology, University of Washington, Seattle, Washington, United States of America; Maastricht University, Netherlands

## Abstract

Skeletal muscle generation of reactive oxygen species (ROS) is increased following contractile activity and these species interact with multiple signaling pathways to mediate adaptations to contractions. The sources and time course of the increase in ROS during contractions remain undefined. Confocal microscopy with specific fluorescent probes was used to compare the activities of superoxide in mitochondria and cytosol and the hydrogen peroxide content of the cytosol in isolated single mature skeletal muscle (*flexor digitorum brevis*) fibers prior to, during, and after electrically stimulated contractions. Superoxide in mitochondria and cytoplasm were assessed using MitoSox red and dihydroethidium (DHE) respectively. The product of superoxide with DHE, 2-hydroxyethidium (2-HE) was acutely increased in the fiber cytosol by contractions, whereas hydroxy-MitoSox showed a slow cumulative increase. Inhibition of nitric oxide synthases increased the contraction-induced formation of hydroxy-MitoSox only with no effect on 2-HE formation. These data indicate that the acute increases in cytosolic superoxide induced by contractions are not derived from mitochondria. Data also indicate that, in muscle mitochondria, nitric oxide (NO) reduces the availability of superoxide, but no effect of NO on cytosolic superoxide availability was detected. To determine the relationship of changes in superoxide to hydrogen peroxide, an alternative specific approach was used where fibers were transduced using an adeno-associated viral vector to express the hydrogen peroxide probe, *HyPer* within the cytoplasmic compartment. *HyPer* fluorescence was significantly increased in fibers following contractions, but surprisingly followed a relatively slow time course that did not appear directly related to cytosolic superoxide. These data demonstrate for the first time temporal and site specific differences in specific ROS that occur in skeletal muscle fibers during and after contractile activity.

## Introduction

Reactive oxygen species (ROS) are generated continuously by skeletal muscle cells and this process is widely recognised to be increased with contractile activity [1]. The primary reactive oxygen and nitrogen species generated by skeletal muscle are superoxide and nitric oxide [1], [2], but there is an on-going discussion about the specific sub-cellular sites that contribute to superoxide generation in muscle during contractions. ROS interact with multiple signalling processes including pathways that mediate adaptive and potentially protective processes in skeletal muscle, such as the transcription factors AP-1 and NF-κB [3]. An increase in superoxide and NO can also lead to oxidative damage, through the formation of highly reactive secondary species such as peroxynitrite and hydroxyl radical [4]–[7].

The lack of any definitive conclusion concerning the sub-cellular sources of ROS that are active during contractions has been in part due to a lack of definitive analytical approaches to study the processes in situ. There are multiple potential approaches to monitoring ROS including electron paramagnetic resonance [8], monitoring of secondary products of ROS reaction with proteins, lipids and DNA [4] and fluorescence microscopy utilising ROS sensitive probes [9]–[11]. Fluorescence microscopy offers the potential to obtain real-time measurements from living cells, but has been severely hampered by the lack of specificity of the probes that are widely available. MitoSox Red and dihydroethidium (DHE) which localise predominantly to the mitochondrial and cytoplasmic compartments of cells respectively [12]–[14] are commonly used probes to detect intracellular superoxide. DHE accumulates in the cell cytoplasm by diffusion and following oxidation the product binds with negatively charged DNA that results in enhanced fluorescence and facilitates indirect superoxide detection. MitoSox is DHE with a triphenylphoshonium cation that facilitates its preferential accumulation (100–1000-fold) and retention within mitochondria [15]. Following oxidation the product also interacts with mitochondrial DNA and RNA with enhanced fluorescence. Many previous studies have monitored non-specific ethidium fluorescence as an index of superoxide activity following loading of cells with DHE or MitoSox, but recent studies have identified 2-hydroxyethidium (2-HE) and the equivalent hydroxylated product of MitoSox (hydroxy-MitoSox) as specific products of the reaction of DHE or MitoSox with superoxide [16], [17]. Analysis of the fluorescence spectra of DHE, MitoSox and their oxidation products has identified a specific excitation wavelength for monitoring hydroxy-MitoSox and 2-HE. The fluorescent detection of the superoxide specific products for both MitoSox and DHE has been reported to require excitation at 396 nm whereas non-specific oxidation products are detected using an excitation of 510 nm with emissions at either excitation monitored at wavelengths >560 nm [12], [15].

Previous approaches to assess hydrogen peroxide within muscle fibers and other cells have primarily used DCFH (2′, 7′ dichlorodihydrofluorescein), but despite its extensive use the lack of specificity of this probe is widely recognised and it has been shown to be sensitive to a number of ROS and reactive nitrogen species (RNS) including superoxide, nitric oxide and peroxynitrite in addition to hydrogen peroxide [18], [19]. The genetically encoded probe, *HyPer* is a circularly permuted yellow fluorescent protein (cpYFP) inserted into the regulatory domain of the specific prokaryotic hydrogen peroxide-sensing protein, OxyR [20] that has been developed to provide a specific probe for hydrogen peroxide, but cells must be transfected with this probe. In the current study isolated muscle fibers were transduced using an adeno-associated virus to express *HyPer* within the cytoplasmic compartment.

Although older data indicated that mitochondria are important in generation of ROS in skeletal muscle following contractions [1], [2], more recent studies have identified a potential role for NADPH oxidases (Noxs) in this process [21]–[23]. Skeletal muscle has been shown to express Nox2 [21] and Nox4 [24] and the Nox4 isoform appears to be present in muscle mitochondria [22], hence these studies also do not define the key sub-cellular sites for ROS generation during contractions. The aims of this study were therefore to use specific approaches to monitor the activities of superoxide in mitochondria and cytosol of isolated mature skeletal muscle fibers prior to, during, and after periods of contractile activity compared with fibers at rest. The relationship of superoxide activity to hydrogen peroxide content of fibers was also examined following transfection of fibers with the hydrogen peroxide-specific probe, *HyPer*.

## Materials and Methods

### Mice

All experiments were performed in accordance with UK Home Office guidelines and under the UK Animals (Scientific Procedures) Act 1986. The protocol was approved by the University of Liverpool animal ethics committee. Male adult (10–16 months old) C57Bl/6 mice were used for all experiments. Mice were humanely killed and the *flexor digitorum brevis* (FDB) muscles were rapidly removed for isolation of single fibers.

### Isolation of single mature skeletal muscle fibers

**Figure 1:**
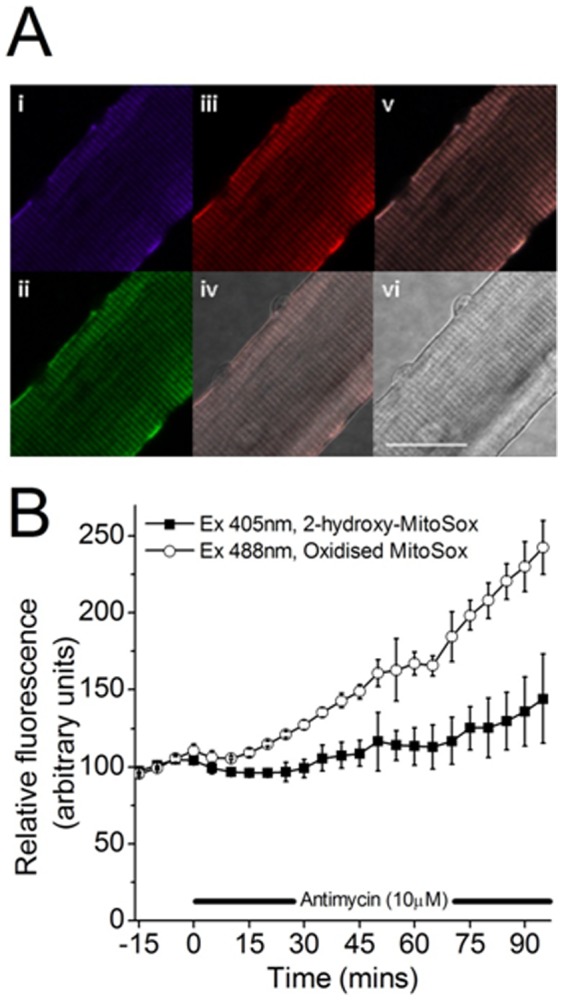
(A) Fluorescent confocal images of a single isolated FDB muscle fiber loaded with MitoSox red (125 nM) and Mitotracker green (20 nM). (i)Fluorescent image of fiber loaded with mitotracker, (ii) Fluorescent image of fiber loaded with MitoSox obtained using excitation at 405 nm, (iii) Fluorescent image of fiber loaded with MitoSox obtained using excitation at 488 nm, (iv) merge of bright field and fluorescent images, (v) merged fluorescent images i–iii, (vi) bright field image. Scale bar = 25 µm. (B) Fluorescence values from MitoSox red loaded fibers (n = 3) with time, prior to -, and after exposure to 10 µM antimycin A (denoted by the black bar). Data from excitation at 405 nm is claimed to reflect 2-hydroxy MitoSox and excitation at 488 nm monitors non-specific oxidation products of MitoSox.

Single fibers were isolated from the FDB muscles of mice using the method of Shefer & Yablonka-Reuveni [25] as modified by Palomero et al., [9]. FDB muscles were incubated for 1.5 hr at 37°C in 0.4% (w/v) Type I collagenase (EC 3.4.24.3, Sigma Chemical Co., Poole, Dorset, UK) in minimum essential medium eagle (MEM) media containing 2 mM glutamine, 50 i.u. penicillin, 50 µg ml^−1^ streptomycin and 10% foetal bovine serum (FBS, Sigma Chemical Co.). The muscles were agitated every 30 minutes during the digestion period. Single myofibers were released by gentle trituration with a wide-bore pipette and fibers were washed three times in MEM media containing 10% FBS. Fibers were plated onto pre-cooled 35 mm glass bottomed cell culture dishes (MatTek, Massachusetts, USA) pre-coated with Matrigel (BD Biosciences, Oxford, UK) in 2.5 ml MEM media containing 10% FBS. Fibers were incubated for 20 hrs at 37°C in a 5% CO_2_ tissue culture incubator. Fibers prepared and cultured in this manner are viable for up to 6 days in culture [9] although in practise all fibers were studied within 30 hours. Experiments were performed on fibers that retained good morphology and exhibited prominent cross-striations (see Figure 1).

### Solutions

MitoSox red and DHE (Invitrogen, Paisley, UK) were diluted in DMSO prior to use. MEM, D-PBS, Apocynin, NG-Nitro-l-arginine methyl ester hydrochloride (L-NAME) and allopurinol were from the Sigma Chemical Co. MEM solution was made of (in mM) MgSO_4_.H_2_O (0.8), KCl (5.4), NaCl (116.4), NaH_2_PO_4_.H_2_O (1), D-glucose (5.5), NaHCO_3_ (26.2), HEPES (10), CaCl_2_.2H_2_O (1.9) and pH 7.4.

### Use of MitoSox red to monitor mitochondrial superoxide in isolated fibers

Fibers were loaded in 2 ml Dulbecco's phosphate-buffered saline (D-PBS) containing 125 nM MitoSox red for 10 minutes at 37°C in a tissue culture incubator. Fibers were then washed twice with D-PBS and two further washes with MEM solution. Fibers were maintained in 2 ml MEM solution during the experimental protocol. Where L-NAME was used to inhibit nitric oxide synthases [26], this was added 30 minutes prior to the commencement of the experiment and maintained throughout the experiment.

### Use of dihydroethidium to monitor cytoplasmic superoxide in isolated fibers

Fibers were loaded by incubation in 2 ml D-PBS containing 5 µM DHE for 20 minutes at 37°C in a tissue culture incubator. Fibers were then washed twice with D-PBS and two further washes with MEM solution. Fibers were maintained in 2 ml MEM solution during the experimental protocol.

### Use of *HyPer* to specifically monitor hydrogen peroxide in isolated fibers

Cyto-*HyPer* obtained from *Evrogen* (Moscow, Russia) was cloned into a recombinant adeno-associated viral vector (pseudotype 6; rAAV6) to generate vector particles that were used for transduction of isolated muscle fibers prior to experimentation. Preparation of rAAV6 vectors was performed essentially as described previously [27]. Fibers were exposed to 7.5×10^9^ adeno-associated viral particles containing the cyto-*HyPer* immediately after isolation and maintained in the presence of the particles for 72 hours in a tissue culture incubator. Fibers then washed three times and maintained in 2 ml MEM-solution during the experimental protocol.

### Confocal microscopy

A Nikon E-Ti inverted microscope with an Okolab heated stage insert to a motorised stage (TI-S-EJOY, Nikon) for a 35 mm petri dish equipped with a C1 confocal (Nikon Instruments Europe BV, Surrey, UK) was used. The confocal had 3 lasers, a diode (UV) with 405 nm excitation, argon with 488 nm excitation and a helium-neon with 543 nm excitation. Acquisition software was EZC1 V.3.9 (12 bit). For assessment of 2-HE and hydroxy-MitoSox, excitation was at 405 nm with the emission collected through a 605/15 filter. For the non-specific (ethidium) products of DHE and MitoSox oxidation, excitation was at 488 nm with the emission collected through a 605/15 filter. To examine cytoplasmic DHE, an additional scan was undertaken (at 405 nm excitation) and the emission was collected through a 450/35 filter. Cyto-*HYPER* was sequentially excited at 405 and 488 nm with emission collected between 515/30 nm, with the ratio of 488/405 nm excitation being presented. Bright field images were acquired using the 543 nm laser. The objective was a PlanApo VC ×60A/1.2NA/0.27 mm working distance water immersion. Pinhole size was 150 µm with a 1.86 µsec pixel dwell time in all cases. Regions of interest for determination of fluorescence/area were selected as previously reported [10]. All experiments were performed at 25°C.

### Electrical stimulation of muscle contraction in isolated fibers

Single muscle fibers were subjected to electrical field stimulation (Harvard Apparatus, Kent, UK) in 35 mm petri dishes using platinum electrodes (Advent, Oxford, UK) as previously described [28]. The total incubation time for the fibers was for 60 minutes with confocal images captured every ten minutes. Following an initial 10 minutes at rest, fibers were stimulated to contract for 10 minutes with trains of bipolar square wave pulses of 2 ms in duration for 0.5 sec repeated every 5 sec at 50 Hz and 30 V/well. Fibers then remained at rest for 20 minutes followed by a second identical stimulation protocol and a final 10 minute recovery period at rest.

### Statistical Analysis

Data are presented as means ± SE for each experiment, where n represents number of mice. Data were analysed using SPSS V.17. Data distribution was checked using KS-test and were normal throughout, variance was checked using Levene's test. Data were then examined by General linear models repeated measures examining treatments (i.e. exposure to drug and/or exposure to contraction), followed by one way ANOVA or Student's t test. Data were considered significant at P<0.05.

## Results

### Detection of superoxide in mitochondria of skeletal muscle fibers using MitoSox red

MitoSox red was used to detect superoxide within mitochondria of single muscle fibers. In initial experiments, fibers were loaded with both MitoSox red and mitotracker green (20 nM) to determine whether MitoSox co-localised with mitotracker (Figure 1A) The ability of MitoSox red in fibers to detect superoxide within mitochondria was confirmed by use of antimycin A, an inhibitor of the electron transport chain that induces generation of superoxide from complex III [29]. Figure 1B shows changes in fluorescence from MitoSox-loaded muscle fibers monitored using two excitation wavelengths, 405 and 488 nm (n = 3). Fibers were exposed to antimycin A (10 µM) at 0 minutes, and throughout the rest of the experiment. After 90 minutes exposure to antimycin A the MitoSox fluorescence had increased by 44±28% when monitored at 405 nm excitation and 143±17% at 488 nm excitation. Best fit slopes of linear lines fitted to the MitoSox fluorescence at the 2 wavelengths between 0 to 95 minutes were significantly different with the slope from fluorescence with 488 nm excitation being approximately 3× steeper than that obtained with 405 nm excitation (slopes of 1.44 versus 0.44 arbitrary units respectively, t-test, P = 0.04). Thus exposure to antimycin A induced oxidation of MitoSox that was detectable using the non-specific excitation wavelength and by analysis of hydroxy-MItoSox albeit at a lower sensitivity. For all subsequent experiments, results using 405 nm excitation (i.e. 2-HE or hydroxy-MitoSox) are presented.

### Detection of superoxide in mitochondria from resting and contracted skeletal muscle fibers using MitoSox red

**Figure 2:**
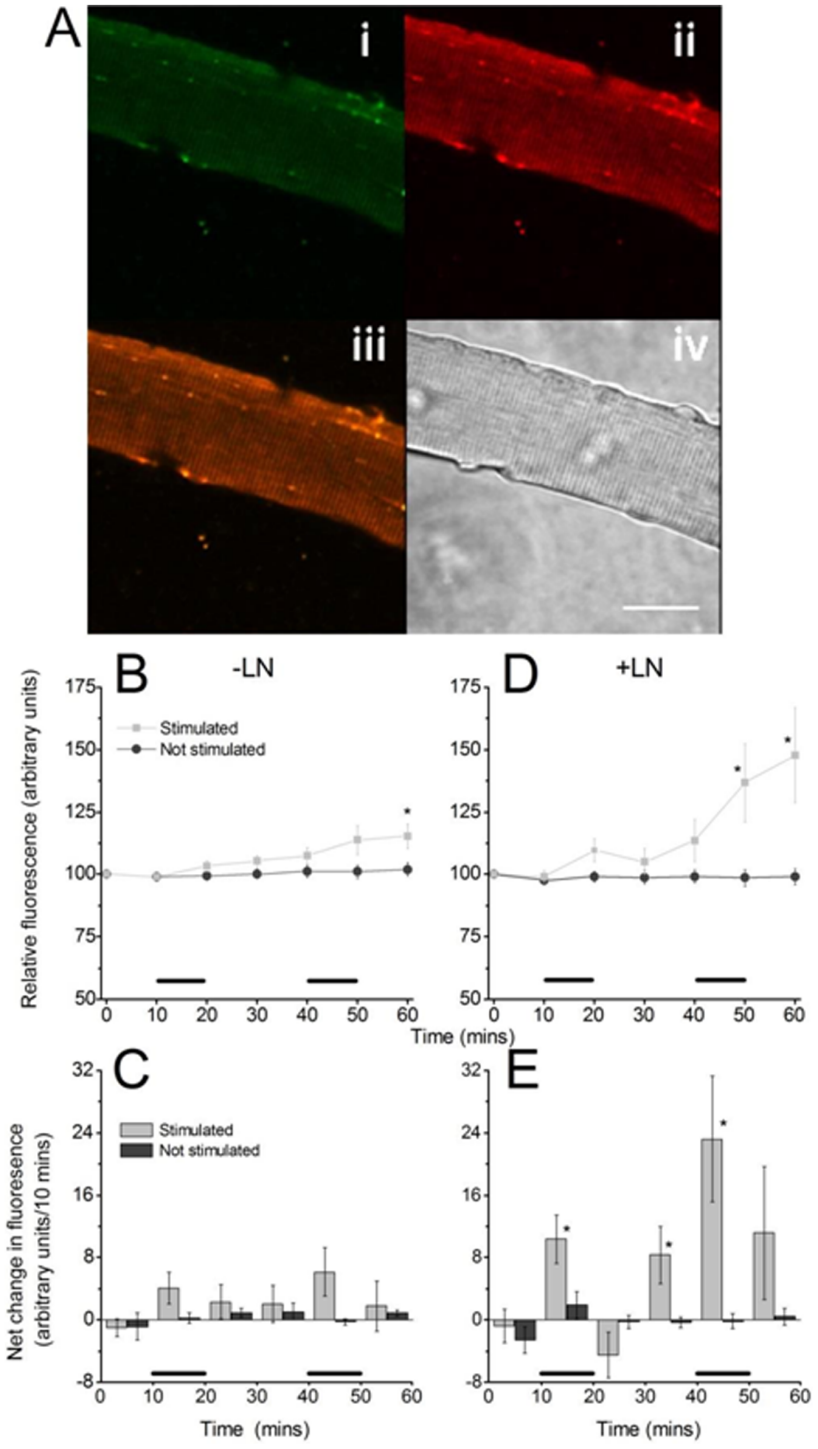
(A) MitoSox red loaded single isolated skeletal muscle fiber, (i) Fluorescent image obtained using excitation at 405 nm, (ii) Fluorescent image obtained using excitation at 488 nm, (iii) merged images i and ii, (iv) bright field image, scale bar = 25 µm. Figures B and D show the relative fluorescence with time from skeletal muscle fibers loaded with MitoSox red (data presented from 405 nm excitation). Fibers were either non-stimulated or subjected to two periods of electrically stimulated contractions during the time periods denoted by a black bar. No significant effect of stimulation over the whole time course was found in the absence of L-NAME (Fig. 2B) whereas in the presence of L-NAME (Fig. 2D) a significant effect of stimulation was found (repeated measures, F = 5.3, P = 0.04) compared with non stimulated fibers. Figures C and E show the rate of change in relative fluorescence (derived from figures B and D ) between the indicated time points with the stimulation periods denoted by black bars. Data shown in Figures B and C were from untreated fibers, data in Figures D and E were from fibers in the presence of 100 µM L-NAME. *P<0.05 compared with non-stimulated fibers at the same time point (n = 6–7 for all groups).

Changes in mitochondrial superoxide were investigated using MitoSox red in resting, non-stimulated single FDB fibers and fibers that were electrically stimulated to contract (Figure 2). Fiber contraction was stimulated by two protocols separated by twenty minutes at rest. The timing of contraction periods are shown by bars on the Figure. Data are presented as the relative fluorescence normalised to the initial fluorescence value (Figures 2B and D) and as the change in fluorescence compared with non-stimulated fibers over each 10 minute period (Figures 2C and E). Figure 2B shows the hydroxy-MitoSox fluorescence changes over time from fibers that were not stimulated to contract and fibers exposed to the two contraction protocols (n = 6 for both groups). Non-stimulated fibers showed no significant increase in hydroxy-MitoSox fluoresecence over time. Stimulated fibers showed a small increase in hydroxy-MitoSox fluorescence over the course of the experiment and by the end a 15±5% increase in fluorescence from these fibers was seen. This change in fluorescence only achieved statistical significance at the final time point compared with values from non-stimulated fibers (one way ANOVA, P = 0.04). Repeated measures analysis over the whole time course showed no significant effect of stimulation between the two groups (F = 4.2, P = 0.069, General linear model). The net change in hydroxy-MitoSox fluorescence did not differ significantly between stimulated and non-stimulated fibers at any time point (Figure 2C).

Since superoxide can rapidly react with NO to form peroxynitrite and hence excess NO might limit superoxide availability for reaction with MitoSox, the effect of the NOS inhibitor, L-NAME, on MitoSox fluorescence was examined (Figures 2D and E). In the presence of L-NAME, the hydroxy-MitoSox fluorescence from non-stimulated fibers was unchanged, but there was an overall increase in the MitoSox fluorescence from stimulated fibers that reached statistical significance after the second contraction protocol and showed a 37±16% increase above non-stimulated values (P = 0.028, one way ANOVA). Analysis over the whole time course showed a significant effect of stimulation (F = 5.3, P = 0.04, General linear model repeated measures) between the stimulated and non-stimulated groups in the presence of L-NAME. Analysis of the rate of change in hydroxy-MitoSox fluorescence in the presence of L-NAME showed increases during the first and second periods of muscle contraction and during the resting period30–40 minutes compared with non-stimulated fibers at the same time points.

### Detection of superoxide in the cytoplasm of resting and contracted skeletal muscle fibers using DHE

**Figure 3:**
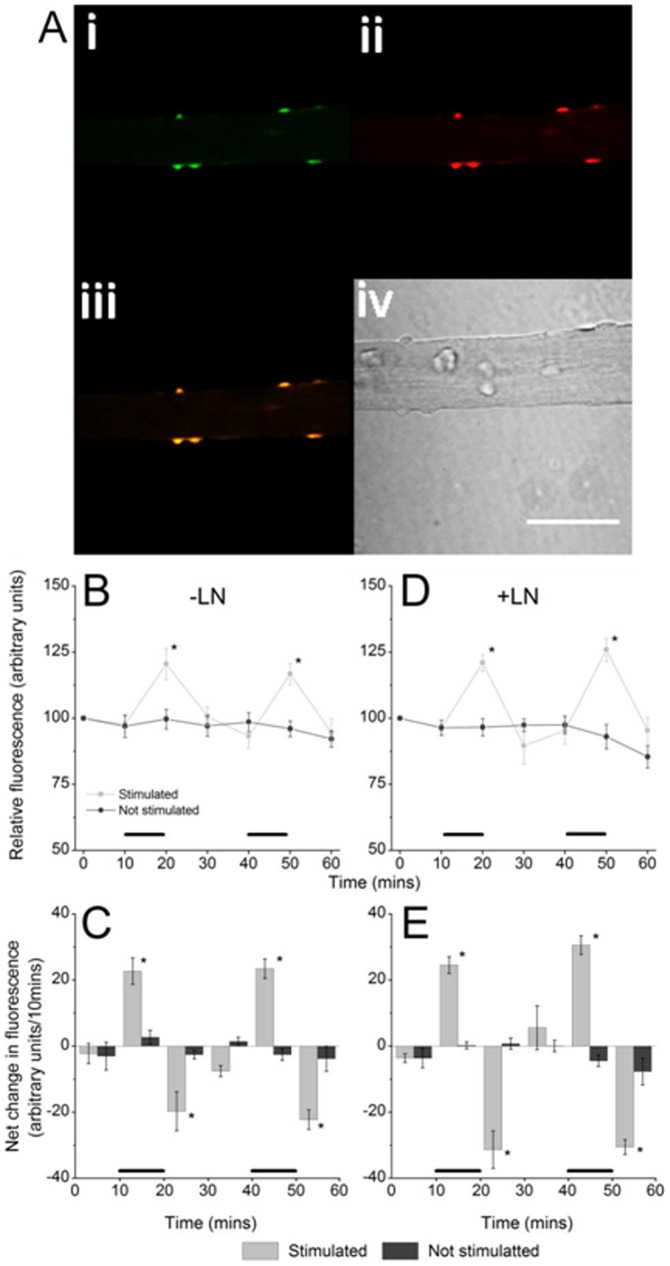
(A) DHE loaded single muscle fiber, (i) Fluorescent image obtained using excitation at 405 nm, (ii) Fluorescent image obtained using excitation at 488 nm, (iii) merged images i and ii, (iv) bright field image, scale bar = 50 µm. Figures B and D show relative fluorescence with time from skeletal muscle fiber nuclei (data presented from 405 nm excitation only, n = 8). Fibers were either at rest or subjected to muscle contraction as denoted by the black bar. Figures C and E show the rate of change in relative fluorescence (derived from figures B and D respectively) between indicated time points. Data shown in Figures B and C were from untreated fibers, data in Figures D and E were from fibers in the presence of 100 µM L-NAME. *P<0.05 compared with non-stimulated fibers at the same time point.

DHE was used to detect superoxide within the cytoplasm of non-stimulated and stimulated muscle fibers in the presence and absence of L-NAME (Figure 3). Fluorescence was monitored from nuclei and showed a significant effect of stimulation with respect to time (repeated measures F = 6.6, P = 0.016). No significant effect of L-NAME on fluorescence from non-stimulated or stimulated fibers was seen. The fluorescence from 2-HE increased acutely and significantly in response to each of the two periods of muscle contraction, exhibiting a 20±6% and 17±4% increase above baseline in the first and second contraction periods in the absence of L-NAME when compared with non-stimulated fibers at the same time points (Figure 3B, one way ANOVA P<0.02 for first and second contractions). Very similar data were obtained in the presence of L-NAME (Figure 3D).

The net change in 2-HE fluorescence (Figure 3C) from the nuclei of non-stimulated fibers showed a significant increase with each period of stimulation (1^st^ stimulation P = 0.01, 2^nd^ stimulation P = 0.01, Student's t-test) compared with non-stimulated fibers. In the 10 minute time period following each stimulation there was significant reversal in the rate of change of fluorescence in the stimulated fibers, leading to a net decline in fluorescence values (after 1^st^ stimulation P = 0.02; after 2^nd^ stimulation P = 0.006, Student's t-test). An identical pattern of changes was observed in the presence of L-NAME (Figure 3E).

### Changes in the muscle fiber content of cytoplasmic DHE during contractions

**Figure 4:**
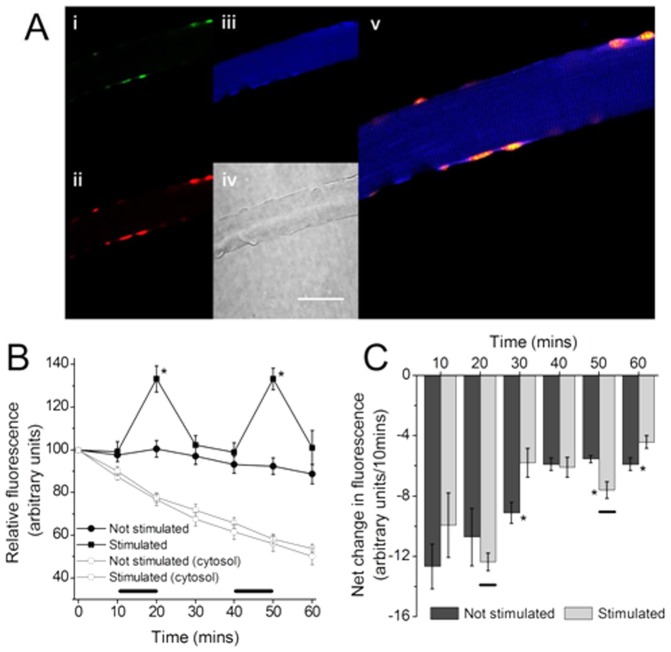
(A) DHE loaded single muscle fiber, (i) Fluorescent image obtained using excitation at 405 nm, (ii) Fluorescent image obtained using excitation at 488 nm, (iii) Fluorescent image obtained with 405 nm emission and 450/35 nm emission monitoring non-oxidised DHE, (iv) bright field image, scale bar = 50 µm, (v) merged figures i–iii. (B) Data from muscle fibers that were either stimulated (as denoted by black bar) or non-stimulated (n = 6 for all groups). Solid symbols show 2-HE fluorescence over time measured from nuclei showing the acute increases in fluorescence following contractions and open symbols show the “unoxidised” DHE fluorescence measured from the same muscle fibers. Cytosolic fluorescence from “unoxidised” DHE decreased by approximately 50% over sixty minutes in both experimental groups reflecting loss of DHE from the cell. (C) Rate of change in relative fluorescence with time for cytosolic DHE (derived from figure B). *P<0.05, compared with non stimulated fibers at the same time point.

The data in Figure 3 show increased nuclei fluorescence resulting from the contractions that was rapidly followed by a decrease in fluorescence to near baseline values. To find a potential explanation for these changes we repeated the experiments with additional measurements of non-oxidised cytoplasmic DHE (see Figure 4Aiii) in parallel with the changes in nuclear 2-HE fluorescence (Figure 4Ai). A potential explanation for the decline in fluorescence signal was that oxidised DHE, which exhibits a 20–40 fold increase in fluorescent intensity upon binding to DNA, might be displaced from DNA resulting in a decline in fluorescence. Because of the proportional increase in fluorescence that occurs on binding to DNA, a decline in signal might result from a modest amount of oxidised DHE being displaced from DNA. Figure 4B shows the pattern of changes in 2-HE measured from the nuclei of contracted fibers in comparison with the total content of DHE from the cytoplasm of the same cells (open symbols) The apparent rates of loss of DHE from fibers were generally similar between stimulated and non-stimulated fibers, but there were small increases in the rate of loss of non-oxidised DHE during the stimulation periods (potentially due to additional loss of DHE to an oxidised form which binds to DNA) followed by small but significant declines in the rate of loss of DHE in the periods immediately following the stimulations (Figure 4C) which we speculate reflects an effect due to DHE being displaced from DNA.

### Detection of hydrogen peroxide in the cytoplasm of resting and contracted skeletal muscle fibers using *HyPer*

**Figure 5:**
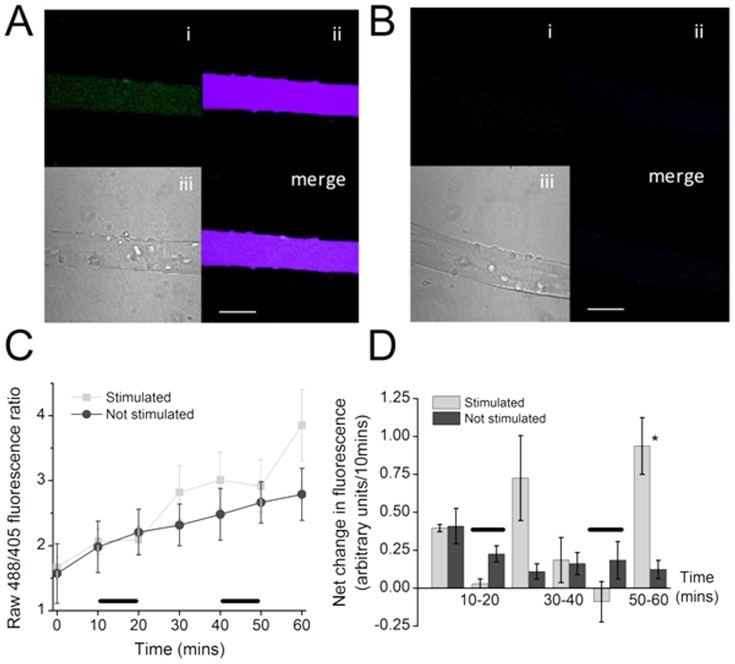
(A) Cyto-*HyPer* transfected single muscle fiber, (i) Fluorescent image obtained using excitation at 405 nm, (ii) Fluorescent image obtained using excitation at 488 nm, (iii) bright field image, (iv) merged images i and ii, scale = 50 µm. (B) Non-transfected single muscle fiber, (i) Fluorescent image obtained using excitation at 405 nm, (ii) Fluorescent image obtained using excitation at 488 nm, (iii) bright field image, (iv) merged images i and ii, scale = 50 µm. (C) Ratio of fluorescence values at excitations of 488/405 nm from fibers transfected with *HyPer* that were either stimulated (as denoted by black bars) or non- stimulated (n = 7 for both groups). (D) Rate of change in ratio of fluorescence at excitations of 488/405 nm from fibers transfected with *HyPer*. *P<0.05, compared with non stimulated fibers at the same time point.

Transduction of fibers with a rAAV6 vector [27] expressing cyto-*HyPer* (Evrogen, Moscow) led to the expression of *HyPer* within the cytoplasm of all fibers. Figure 5A shows the distribution of *HyPer* in a single fiber at 72 hours post-transduction and demonstrates the predominant localisation to the cytoplasmic compartment. Figure 5B shows the non-specific fluorescence from control fibers that were not transfected with *HyPer*, but exposed to the contraction protocols. In *HyPer* transfected fibers fluorescence was generally increased in the contracted fibers (Figure 5C), but the rate of change in fluorescence was only significantly increased in the contracted fibers during the time periods immediately after cessation of contractions (Figure 5D).

## Discussion

The potential for skeletal muscle to generate reactive oxygen and nitrogen species in response to contractile activity has been widely examined, but despite extensive descriptive studies there remains considerable debate about the nature and time-course of changes in different cellular compartments [1], [2]. Inhibitor and other studies have recently indicated that non-mitochondrial sources may be important [21]–[23], but recent developments in approaches to localise ROS-sensitive probes within sub-cellular compartments of cells and to improve the specificity of probes provide the opportunity to directly address these questions. The current study has demonstrated that cytosolic superoxide activity increases acutely in response to contractions, but this was not accompanied by a similar acute change in mitochondrial superoxide availability and that modification of NO availability only influenced mitochondrial superoxide with no effect on cytosolic superoxide. Surprisingly, changes in cytosolic hydrogen peroxide monitored using the *HyPer* probe were found to follow a considerably slower time course than the rapid changes in cytosolic superoxide observed following contractions.

Loading of cells with DHE and monitoring of its oxidation through measurement of ethidium fluorescence has been used for a considerable time as an index of superoxide activity [14], [30], [31], but more recent studies have identified 2-hydroxyethidium (2-HE) as a specific product of the reaction of DHE with superoxide [12], [15], [16]. Monitoring of 2-HE has been described through analysis by HPLC techniques [11], [13], [16], [32] or through selective excitation of the 2-HE product in fluorescence microscopy [12], [15]. DHE and MitoSox are structurally similar, the triphenyl-phosphonium cation being incorporated into MitoSox to facilitate its charge dependant accumulation in mitochondria. The superoxide specific product of MitoSox oxidation has also been reported to be detected by selective excitation of the hydroxy-MitoSox product in fluorescence microscopy in an analogous manner to 2-HE [12], [15]. The data shown in Figure 1 confirms the localisation of MitoSox to the mitochondria of isolated mature muscle fibers. MitoSox red fluorescence was found to co-localise with mitotracker green (Figure 1A(v)) confirming the mitochondrial selectivity and no MitoSox fluorescence was seen from nuclei (Figure 1A(ii–iii) respectively). In contrast, the distribution of DHE-oxidation products in DHE loaded fibers was predominantly nuclear (Figure 3A(i–ii)). Although DHE is able to enter mitochondria, it does not accumulate and predominantly remains cytosolic. Following oxidation of DHE, the products intercalate into nuclear DNA and fluorescence is measured from nuclei. The data in Figure 1 also show the relative insensitivity of monitoring of 2-HE (with excitation at 405 nm) compared with the monitoring of non-specific oxidation products with 488 nm excitation. Nevertheless changes in the specific product were detectable following treatment with antimycin A and this approach was used for all further studies.

A comparison of the pattern of formation of 2-HE in the cytosol of DHE loaded fibers (Figure 3) with hydroxy-MitoSox in the mitochondria of MitoSox-loaded fibers (Figure 2) showed that cytosolic superoxide increased acutely in response to contractile activity but there was no similar acute increase in hydroxy-MitoSox in response to contractions. In addition treatment of fibers with the NOS inhibitor, L-NAME significantly increased the formation of hydroxy-MitoSox, but had no effect on 2-HE formation. Taken together, these data do not support the possibility that contractile activity induces an increase in mitochondrial superoxide generation that results in a subsequent increase in cytosolic superoxide. Skeletal muscle is reported to contain both the type I (neuronal) and type III (endothelial) NOS isoforms with eNOS localised to mitochondria [33]. Contraction increases the production of NO by muscle fibers [2], [10], [34] that is reported to occur primarily through activation of nNOS [10]. The data presented here indicate that non-specific inhibition of NOS using L-NAME had a significant effect on superoxide availability in mitochondria, but no effect on the cytosolic superoxide implying that the effect is likely to be due to an effect on mitochondrial eNOS [33], but this cannot be confirmed by the current data.

The rapid decline in 2-hydroxyethidium fluorescence that occurred following the end of the contraction protocols is difficult to explain since the fluorescent oxidised products of DHE should not degrade rapidly. Data in Figure 4 suggest that this may be due to some dislocation of the oxidised DHE from nuclei and since the fluorescence from DHE oxidation products is increased markedly by binding to DNA, a relatively small dislocation of these products from DNA might produce the effect observed in the period following the end of contractions.

The overall conclusion that mitochondria are not the major source of the acute contraction-induced increase in superoxide is supported by previous studies using MitoSox which assessed the non-specific oxidation products [35] and by measurements of mitochondrial redox potential monitored using mito-roGFP (a mitochondrial targeted redox sensitive GFP) [36]. A number of recent studies have focussed on the potential role of NADPH oxidases as a source of superoxide generation during contractions. This possibility is supported by the presence of Nox2 [21] and Nox4 [24] in skeletal muscle, the demonstration that inhibition of Noxs can reduce contraction-induced generation of ROS [22] and the direct demonstration of activation of Nox2 by electrical stimulation of single FDB fibers [23]. There is also some evidence that Nox4 is found in muscle mitochondria [22], but the data presented here argue against Noxs localised to mitochondria playing a role in the contraction induced generation of superoxide.

A further aspect of the data obtained was the gradual increase in hydroxy-MitoSox fluorescence that was seen to occur with time both in the absence or presence of L-NAME. This increase appeared to be initiated by contractile activity, but unlike the changes in cytosolic 2-hydroxyethidium, the hydroxy-MitoSox fluorescence also increased during some periods when contraction was not occurring. We speculate that these changes in hydroxy-MitoSox reflect a general increase in mitochondrial activity that is stimulated by the contractions, but occurs over a slower and delayed time course. This may be highly relevant to understanding ROS generation during studies of longer term exercise or training programmes and recognition that acute and longer term contraction protocols may activate different sources of superoxide generation could explain several apparent contradictions in the literature [1].

Previous attempts to monitor hydrogen peroxide in muscle fibers have primarily used DCFH or other non-specific probes [19], [35]. *HyPer* is a genetically encoded specific probe for hydrogen peroxide and this was expressed in single isolated muscle fibers using an adeno-associated viral vector [27], [37]. The *HyPer* construct that was used localises the protein to the cytosol and the data in Figure 5 show slow increases in *HyPer* fluorescence occur over a time course that does not follow those seen from 2-hydroxyethidium or hydroxy-MitoSox or reflect the time course seen in previous data obtained with the non-selective probe, DCFH [9]. The *HyPer* probe is reversible and a decline in fluorescence reflects reduction by endogenous cellular thiols [20], [38]. The kinetics of *HyPer* responses to hydrogen peroxide have been studied by Markincheva et al [38] who reported the half oxidation time of *HyPer* by hydrogen peroxide to be ∼6 seconds. This is slower than DCFH reaction with multiple ROS, but indicates that the rate of oxidation would not be limiting with the experimental design used here (where fluorescence measurements were taken every 10 minutes). Thus the data obtained appear to reflect a true delay in the increase in fiber hydrogen peroxide concentration in response to contractions. The reasons underlying the slower changes are unclear, but may reflect rapid clearance of hydrogen peroxide through intracellular activities of glutathione peroxidise, catalase etc, or may be associated with differences in cellular distribution between DHE and *HyPer*. An interesting possibility is that since the potential sources for cytosolic superoxide generation, such as NADPH oxidases, are localised to the plasma membrane, the local changes in hydrogen peroxide concentration occur relatively slowly compared with superoxide since hydrogen peroxide can rapidly diffuse across the plasma membrane whereas superoxide cannot.

These data appear to be the first to report specific changes in hydrogen peroxide in muscle fibers resulting from contractile activity and they provide support for the key role that investigators have ascribed to hydrogen peroxide in regulation of multiple cell functions, such as force generation, stress responses and modification of gene expression [1]. An examination of the sensitivity of the *HyPer* probe in muscle fibers to exogenous hydrogen peroxide is provided as supplementary data (Figure S1) and shows that changes in *HyPer* fluorescence from fibers were apparent at ∼5 µM external hydrogen peroxide. This sensitivity is approximately equivalent to that of other probes such as DCFH [9] and demonstrates that the likely increase in intracellular hydrogen peroxide in muscle fibers following contractile activity is likely to be less than 1 µM, a figure that is in line with previous estimates [2].

In summary, the current study has demonstrated that cytosolic superoxide activity increased acutely in response to contractions, but this was not accompanied by a similar acute change in mitochondrial superoxide availability and that inhibition of NOS influenced mitochondrial superoxide availability but not cytosolic availability. These data therefore strongly support the hypothesis that the rise in cytosolic superoxide that accompanies contractile activity in skeletal muscle fibers is not derived from mitochondrial sources. Studies with the specific *HyPer* probe confirm that hydrogen peroxide is generated within muscle fibers, but demonstrate that the acute increase in muscle fiber cytosolic superoxide induced by contractions is not associated with an immediate increase in cytosolic hydrogen peroxide content.

## Supporting Information

Figure S1 Effect of increasing extracellular hydrogen peroxide concentration on the fluorescence monitored at excitations of 488/405 nm from fibers transfected with *HyPer* (n = 4 at each concentration).
(TIF)

## References

[1] Powers SK, Jackson MJ (2008) Exercise-induced oxidative stress: cellular mechanisms and impact on muscle force production. Physiol Rev 88: 1243–1276. 18923182 10.1152/physrev.00031.2007 PMC2909187

[2] Jackson MJ (2011) Control of reactive oxygen species production in contracting skeletal muscle. Antioxid Redox Signal 15: 2477–2486. 21699411 10.1089/ars.2011.3976 PMC3176346

[3] Vasilaki A, McArdle F, Iwanejko LM, McArdle A (2006) Adaptive responses of mouse skeletal muscle to contractile activity: The effect of age. Mech Ageing Dev 127: 830–839. 16996110 10.1016/j.mad.2006.08.004

[4] Halliwell B, Gutteridge JMC (1989) Free radical biology and medicine. Oxford University Press.

[5] Pacher P, Beckman JS, Liaudet L (2007) Nitric oxide and peroxynitrite in health and disease. Physiol Rev 87: 315–424. 17237348 10.1152/physrev.00029.2006 PMC2248324

[6] Vasilaki A, Mansouri A, Remmen H, van der Meulen JH, Larkin L, et al (2006) Free radical generation by skeletal muscle of adult and old mice: effect of contractile activity. Aging Cell 5: 109–117. 16626390 10.1111/j.1474-9726.2006.00198.x

[7] Vasilaki A, Simpson D, McArdle F, McLean L, Beynon RJ, et al (2007) Formation of 3-nitrotyrosines in carbonic anhydrase III is a sensitive marker of oxidative stress in skeletal muscle. Proteomics Clin Appl 1: 362–372. 21136689 10.1002/prca.200600702

[8] Close GL, Ashton T, McArdle A, Maclaren DP (2005) The emerging role of free radicals in delayed onset muscle soreness and contraction-induced muscle injury. Comp Biochem Physiol A Mol Integr Physiol 142: 257–266. 16153865 10.1016/j.cbpa.2005.08.005

[9] Palomero J, Pye D, Kabayo T, Spiller DG, Jackson MJ (2010) In situ detection and measurement of intracellular reactive oxygen species in single isolated mature skeletal muscle fibers by real time fluorescence microscopy. Antioxid Redox Signal 10: 1463–1474. 10.1089/ars.2007.2009 PMC2536563 18407749

[10] Pye D, Palomero J, Kabayo T, Jackson MJ (2007) Real-time measurement of nitric oxide in single mature mouse skeletal muscle fibers during contractions. J Physiol 581: 309–318. 17331997 10.1113/jphysiol.2006.125930 PMC2075220

[11] Sakellariou GK, Pye D, Vasilaki A, Zibrik L, Palomero J, et al (2011) Role of superoxide-nitric oxide interactions in the accelerated age-related loss of muscle mass in mice lacking Cu,Zn superoxide dismutase. Aging Cell 10: 749–760. 21443684 10.1111/j.1474-9726.2011.00709.x PMC3531889

[12] Robinson KM, Janes MS, Pehar M, Monette JS, Ross MF, et al (2006) Selective fluorescent imaging of superoxide in vivo using ethidium-based probes. Proc Natl Acad Sci U S A 103: 15038–15043. 17015830 10.1073/pnas.0601945103 PMC1586181

[13] Zhao H, Kalivendi S, Zhang H, Joseph J, Nithipatikom K, et al (2003) Superoxide reacts with hydroethidine but forms a fluorescent product that is distinctly different from ethidium: potential implications in intracellular fluorescence detection of superoxide. Free Radic Biol Med 34: 1359–1368. 12757846 10.1016/s0891-5849(03)00142-4

[14] Zielonka J, Vasquez-Vivar J, Kalyanaraman B (2008) Detection of 2-hydroxyethidium in cellular systems: a unique marker product of superoxide and hydroethidine. Nat Protoc 3: 8–21. 18193017 10.1038/nprot.2007.473

[15] Robinson KM, Janes MS, Beckman JS (2008) The selective detection of mitochondrial superoxide by live cell imaging. Nat Protoc 3: 941–947. 18536642 10.1038/nprot.2008.56

[16] Fink B, Laude K, McCann L, Doughan A, Harrison DG, et al (2004) Detection of intracellular superoxide formation in endothelial cells and intact tissues using dihydroethidium and an HPLC-based assay. Am J Physiol Cell Physiol 287: C895–902. 15306539 10.1152/ajpcell.00028.2004

[17] Mukhopadhyay P, Rajesh M, Yoshihiro K, Hasko G, Pacher P (2007) Simple quantitative detection of mitochondrial superoxide production in live cells. Biochem Biophys Res Commun 358: 203–208. 17475217 10.1016/j.bbrc.2007.04.106 PMC2228267

[18] Arbogast S, Reid MB (2004) Oxidant activity in skeletal muscle fibers is influenced by temperature, CO2 level, and muscle-derived nitric oxide. Am J Physiol Regul Integr Comp Physiol 287: R698–705. 15178539 10.1152/ajpregu.00072.2004

[19] Murrant CL, Andrade FH, Reid MB (1999) Exogenous reactive oxygen and nitric oxide alter intracellular oxidant status of skeletal muscle fibers. Acta Physiol Scand 166: 111–121. 10383490 10.1046/j.1365-201x.1999.00555.x

[20] Belousov VV, Fradkov AF, Lukyanov KA, Staroverov DB, Shakhbazov KS, et al (2006) Genetically encoded fluorescent indicator for intracellular hydrogen peroxide. Nat Methods 3: 281–286. 16554833 10.1038/nmeth866

[21] Hidalgo C, Sánchez G, Barrientos G, Aracena-Parks P (2006) A transverse tubule NADPH oxidase activity stimulates calcium release from isolated triads via ryanodine receptor type 1 S -glutathionylation. J Biol Chem 281: 26473–2682. 16762927 10.1074/jbc.M600451200

[22] Sakellariou GK, Vasilaki A, Palomero J, Kayani A, Zibrik L, et al (2013) Studies of mitochondrial and nonmitochondrial sources implicate nicotinamide adenine dinucleotide phosphate oxidase(s) in the increased skeletla muscle superoxide generation that occurs during contractile activity. Antiox Redox Signal 18: 603–621. 10.1089/ars.2012.4623 PMC3549212 23050834

[23] Pal R, Basu Thakur P, Li S, Minard C, Rodney GG (2013) Real-time imaging of NADPH oxidase activity in living cells using a novel fluorescent protein reporter. PLoS One 8: e63989. 23704967 10.1371/journal.pone.0063989 PMC3660327

[24] Sun QA, Hess DT, Nogueira L, Yong S, Bowles DE, et al (2011) Oxygen-coupled redox regulation of the skeletal muscle ryanodine receptor-Ca2+ release channel by NADPH oxidase 4. Proc Natl Acad Sci U S A 108: 16098–16103. 21896730 10.1073/pnas.1109546108 PMC3179127

[25] Shefer G, Yablonka-Reuveni Z (2005) Isolation and culture of skeletal muscle myofibers as a means to analyze satellite cells. Methods Mol Biol 290: 281–304. 15361669 10.1385/1-59259-838-2:281 PMC3523695

[26] Pfeiffer S, Leopold E, Schmidt K, Brunner F, Mayer B (1996) Inhibition of nitric oxide synthesis by NG-nitro-L-arginine methyl ester (L-NAME): requirement for bioactivation to the free acid, NG-nitro-L-arginine. Br J Pharmacol 118: 1433–1440. 8832069 10.1111/j.1476-5381.1996.tb15557.x PMC1909689

[27] Blankinship MJ, Gregorevic P, Allen JM, Harper SQ, Harper H, et al (2004) Efficient transduction of skeletal muscle using vectors based on adeno-associated virus serotype 6. Mol Ther 10: 671–678. 15451451 10.1016/j.ymthe.2004.07.016

[28] McArdle A, Pattwell D, Vasilaki A, Griffiths RD, Jackson MJ (2001) Contractile activity-induced oxidative stress: cellular origin and adaptive responses. Am J Physiol Cell Physiol 280: C621–627. 11171582 10.1152/ajpcell.2001.280.3.C621

[29] Muller FL, Liu Y, Van Remmen H (2004) Complex III releases superoxide to both sides of the inner mitochondrial membrane. J Biol Chem 279: 49064–49073. 15317809 10.1074/jbc.M407715200

[30] Peshavariya HM, Dusting GJ, Selemidis S (2007) Analysis of dihydroethidium fluorescence for the detection of intracellular and extracellular superoxide produced by NADPH oxidase. Free Radic Res 41: 699–712. 17516243 10.1080/10715760701297354

[31] Whiteman M, Dogra Y, Winyard PG, Armstrong JS (2009) Detection and measurement of reactive oxygen intermediates in mitochondria and cells. Methods Mol Biol 476: 28–49. 10.1007/978-1-59745-129-1_3 19157007

[32] Zhao H, Joseph J, Fales HM, Sokoloski EA, Levine RL, et al (2005) Detection and characterization of the product of hydroethidine and intracellular superoxide by HPLC and limitations of fluorescence. Proc Natl Acad Sci U S A 102: 5727–5732. 15824309 10.1073/pnas.0501719102 PMC556312

[33] Stamler JS, Meissner G (2001) Physiology of nitric oxide in skeletal muscle. Physiol Rev 81: 209–237. 11152758 10.1152/physrev.2001.81.1.209

[34] Balon TW, Nadler JL (1994) Nitric oxide release is present from incubated skeletal muscle preparations. J Appl Physiol 77: 2519–2521. 7896585 10.1152/jappl.1994.77.6.2519

[35] Aydin J, Andersson DC, Hanninen SL, Wredenberg A, Tavi P, et al (2009) Increased mitochondrial Ca2+ and decreased sarcoplasmic reticulum Ca2+ in mitochondrial myopathy. Hum Mol Genet 18: 278–288. 18945718 10.1093/hmg/ddn355

[36] Michaelson LP, Shi G, Ward CW, Rodney GG (2010) Mitochondrial redox potential during contraction in single intact muscle fibers. Muscle Nerve 42: 522–529. 20730875 10.1002/mus.21724 PMC3015179

[37] Rabinowitz JE, Samulski J (1998) Adeno-associated virus expression systems for gene transfer. Curr Opin Biotechnol 9: 470–475. 9821274 10.1016/s0958-1669(98)80031-1

[38] Markvicheva KN, Bilan DS, Mishina NM, Gorokhovatsky AY, Vinokurov LM, et al (2011) A genetically encoded sensor for H2O2 with expanded dynamic range. Bioorg Med Chem 19: 1079–1084. 20692175 10.1016/j.bmc.2010.07.014

