# Supplementary figures and images for: Skeletal Muscle Contractions Induce Acute Changes in Cytosolic Superoxide, but Slower Responses in Mitochondrial Superoxide and Cellular Hydrogen Peroxide
Source: PLoS One. 2014 May 29;9(5):e96378. doi: 10.1371/journal.pone.0096378 (PMC4038480; doi:10.1371/journal.pone.0096378)

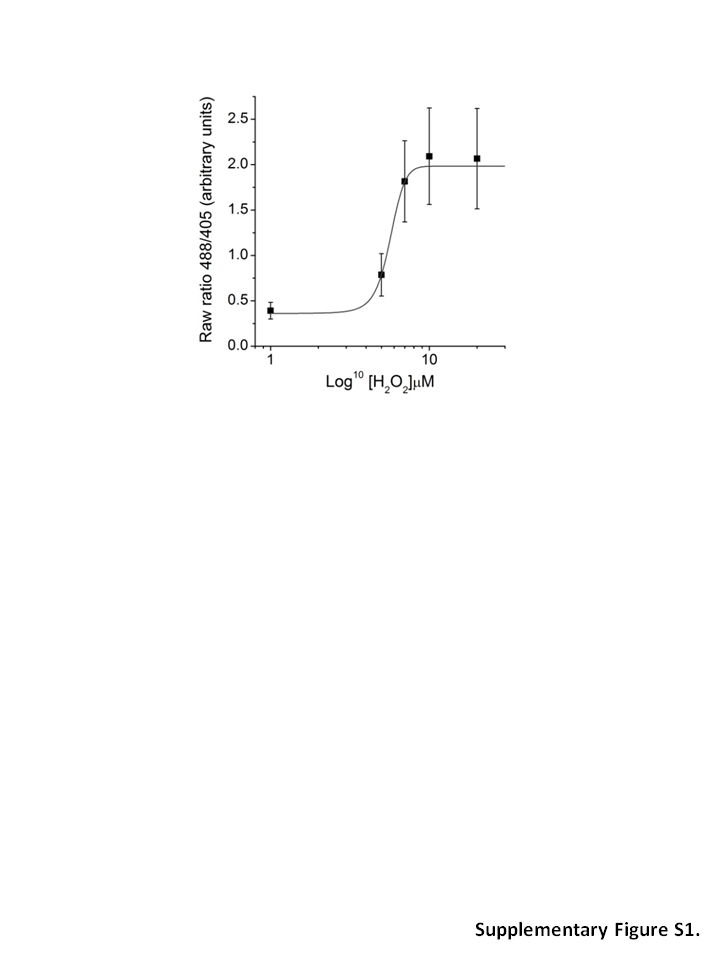

Supplement: Figure S1 — Effect of increasing extracellular hydrogen peroxide concentration on the fluorescence monitored at excitations of 488/405 nm from fibers transfected with HyPer (n = 4 at each concentration). (TIF) [file pone.0096378.s001.tif]
